# Supplementary material for: Validation of prognostic scores predicting mortality in acute liver decompensation or acute-on-chronic liver failure: A Thailand multicenter study
Source: PLoS One. 2022 Nov 22;17(11):e0277959. doi: 10.1371/journal.pone.0277959 (PMC9681104; doi:10.1371/journal.pone.0277959)
Supplement: S1 Table — (DOCX) [file pone.0277959.s001.docx]

**S1 Table. Diagnostic performance of each prognostic score in predicting short-term mortality**

|  | 30-day mortality | | | 90-day mortality | | |
| --- | --- | --- | --- | --- | --- | --- |
|  | p value | OR (95%CI) | AUROC | p value | OR (95%CI) | AUROC |
| ACLF patients | | | | | | |
| - CTP score | <0.001 | 1.22 (1.14-1.30) | 0.610 | <0.001 | 1.29 (1.20-1.38) | 0.642 |
| - MELD score | <0.001 | 1.07 (1.05-1.09) | 0.613 | <0.001 | 1.08 (1.06-1.11) | 0.612 |
| - MELD-Na score | 0.017 | 1.03 (1.01-1.06) | 0.599 | 0.020 | 1.04 (1.01-1.07) | 0.606 |
| - CLIF-OF score | <0.001 | 1.43 (1.32-1.55) | 0.705 | <0.001 | 1.47 (1.35-1.60) | 0.709 |
| - CLIF-C ACLF score | <0.001 | 1.07 (1.05-1.11) | 0.693 | <0.001 | 1.08 (1.05-1.12) | 0.705 |
| - ALBI score | <0.001 | 3.19 (1.67-6.08) | 0.605 | 0.065 | 1.82 (0.93-3.55) | 0.569 |
| AD patients | | | | | | |
| - CTP score | 0.102 | 1.10 (0.98-1.23) | 0.573 | 0.024 | 1.14 (1.02-1.27) | 0.596 |
| - MELD score | 0.551 | 1.01 (0.97-1.06) | 0.536 | 0.081 | 1.04 (1.00-1.08) | 0.582 |
| - MELD-Na score | 0.872 | 1.00 (0.97-1.04) | 0.513 | 0.057 | 1.03 (1.00-1.06) | 0.583 |
| - CLIF-OF score | 0.240 | 1.14 (0.92-1.45) | 0.580 | 0.252 | 1.13 (0.92-1.39) | 0.568 |
| - CLIF-C AD score | 0.194 | 1.02 (0.99-1.04) | 0.547 | 0.024 | 1.03 (1.00-1.06) | 0.588 |
