# Supplementary material for: Validation of prognostic scores predicting mortality in acute liver decompensation or acute-on-chronic liver failure: A Thailand multicenter study
Source: PLoS One. 2022 Nov 22;17(11):e0277959. doi: 10.1371/journal.pone.0277959 (PMC9681104; doi:10.1371/journal.pone.0277959)
Supplement: S2 Table — (DOCX) [file pone.0277959.s002.docx]

**S2 Table. Predictive performance of prognostic scores for mortality among patients with ACLF**

| Prognostic scores | 30-Day mortality | | | | 90-Day mortality | | | |
| --- | --- | --- | --- | --- | --- | --- | --- | --- |
|  | Univariate analysis | | Multivariate analysis | | Univariate analysis | | Multivariate analysis | |
|  | p value | OR (95%CI) | p value | aOR (95%CI) | p value | OR (95%CI) | p value | aOR (95%CI) |
| CLIF-C OF score | <0.001 | 1.43 (1.32-1.55) | 0.010 | 1.26 (1.06-1.51) | <0.001 | 1.47 (1.35-1.60) | 0.023 | 1.23 (1.03-1.56) |
| CLIF-C ACLF score | <0.001 | 1.07 (1.05-1.11) | 0.038 | 1.04 (1.00-1.08) | <0.001 | 1.08 (1.05-1.12) | 0.059 | 1.04 (1.00-1.08) |
| CTP score | <0.001 | 1.22 (1.14-1.30) | 0.543 | 1.03 (0.93-1.15) | <0.001 | 1.29 (1.20-1.38) | 0.054 | 1.14 (1.00-1.33) |
| MELD score | <0.001 | 1.07 (1.05-1.09) | 0.653 | 0.99 (0.95-1.03) | <0.001 | 1.08 (1.06-1.11) | 0.851 | 1.00 (0.96-1.05) |
| ALBI score | <0.001 | 3.19 (1.67-6.08) | 0.063 | 2.12 (0.96-4.69) | 0.08 | 1.82 (0.93-3.55) | 0.534 | 0.73 (0.28-1.95) |
