# Supplementary material for: Validation of prognostic scores predicting mortality in acute liver decompensation or acute-on-chronic liver failure: A Thailand multicenter study
Source: PLoS One. 2022 Nov 22;17(11):e0277959. doi: 10.1371/journal.pone.0277959 (PMC9681104; doi:10.1371/journal.pone.0277959)
Supplement: S3 Table — (DOCX) [file pone.0277959.s003.docx]

**S3 Table. Predictive factors for mortality among patients with ACLF**

| Clinical parameters | 30-Day mortality | | | | 90-Day mortality | | | |
| --- | --- | --- | --- | --- | --- | --- | --- | --- |
|  | Univariate analysis | | Multivariate analysis | | Univariate analysis | | Multivariate analysis | |
|  | p value | OR (95%CI) | p value | OR (95%CI) | p value | OR (95%CI) | p value | OR (95%CI) |
| Age | 0.008 | 1.02 (1.00-1.03) |  |  | 0.004 | 1.02 (1.01-1.03) |  |  |
| Male | 0.550 | 1.11 (0.79-1.57) |  |  | 0.739 | 1.06 (0.75-1.51) |  |  |
| Presence of diabetes mellitus | 0.307 | 0.71 (0.36-1.38) |  |  | 0.086 | 0.53 (0.25-1.10) |  |  |
| Bacterial infection | 0.654 | 0.90 (0.56-1.44) |  |  | 0.595 | 1.16 (0.68-1.97) |  |  |
| ACLF grade | <0.001 | 2.06 (1.77-2.41) | 0.393 | 0.76 (0.40-1.43) | <0.001 | 2.25 (1.89-2.68) | 0.454 | 0.75 (0.36-1.58) |
| Biochemical profiles | | | | | | | | |
| Na | 0.720 | 1.00 (0.97-1.02) |  |  | 0.121 | 0.98 (0.96-1.01) |  |  |
| Hemoglobin | 0.822 | 1.01 (0.96-1.05) |  |  | 0.812 | 1.01 (0.96-1.05) |  |  |
| WBC | 0.055 | 1.00 (1.00-1.00) |  |  | 0.075 | 1.00 (1.00-1.00) |  |  |
| Platelet | 0.235 | 1.00 (1.00-1.00) |  |  | 0.405 | 1.00 (1.00-1.00) |  |  |
| INR | <0.001 | 2.01 (1.58-2.56) | 0.524 | 0.91 (0.69-1.20) | <0.001 | 2.00 (1.53-2.61) | 0.075 | 0.76 (0.57-1.03) |
| Creatinine | <0.001 | 1.48 (1.26-1.73) | 0.038 | 1.27 (1.01-1.59) | <0.001 | 1.61 (1.33-1.94) | 0.063 | 1.30 (0.99-1.72) |
| Bicarbonate | 0.006 | 0.96 (0.93-0.99) | 0.838 | 0.99 (0.94-1.05) | 0.013 | 0.96 (0.94-0.99) | 0.262 | 1.04 (0.97-1.12) |
| Total bilirubin | <0.001 | 1.04 (1.02-1.06) | 0.596 | 1.01 (0.98-1.05) | <0.001 | 1.04 (1.02-1.06) | 0.462 | 1.02 (0.98-1.06) |
| AST | <0.001 | 1.00 (1.00-1.00) |  |  | 0.045 | 1.00 (1.00-1.00) |  |  |
| ALT | 0.001 | 1.00 (1.00-1.00) |  |  | 0.009 | 1.00 (1.00-1.00) |  |  |
| ALP | 0.102 | 1.00 (1.00-1.00) |  |  | 0.073 | 1.00 (1.00-1.00) |  |  |
| Albumin | 0.006 | 0.70 (0.55-0.91) | 0.404 | 0.89 (0.68-1.17) | 0.061 | 0.89 (0.79-1.01) | 0.257 | 0.75 (0.46-1.23) |
| Lactate | <0.001 | 1.09 (1.04-1.14) | 0.415 | 1.03 (0.96-1.10) | 0.013 | 1.06 (1.01-1.12) | 0.235 | 1.05 (0.97-1.15) |
| Prognostic scores | | | | | | | | |
| CLIF-C OF score | <0.001 | 1.43 (1.32-1.55) | 0.018 | 1.44 (1.07-1.94) | <0.001 | 1.47 (1.35-1.60) | 0.037 | 1.48 (1.02-2.13) |
| CLIF-C ACLF score | <0.001 | 1.07 (1.05-1.11) | 0.394 | 1.02 (0.98-1.06) | <0.001 | 1.08 (1.05-1.12) | 0.576 | 1.01 (0.97-1.06) |
| CTP score | <0.001 | 1.22 (1.14-1.30) |  |  | <0.001 | 1.29 (1.20-1.38) |  |  |
| MELD score | <0.001 | 1.07 (1.05-1.09) |  |  | <0.001 | 1.08 (1.06-1.11) |  |  |
| MELD-Na score | 0.017 | 1.03 (1.01-1.06) |  |  | 0.020 | 1.04 (1.01-1.07) |  |  |
