# Supplementary material for: Validation of prognostic scores predicting mortality in acute liver decompensation or acute-on-chronic liver failure: A Thailand multicenter study
Source: PLoS One. 2022 Nov 22;17(11):e0277959. doi: 10.1371/journal.pone.0277959 (PMC9681104; doi:10.1371/journal.pone.0277959)
Supplement: S4 Table — (DOCX) [file pone.0277959.s004.docx]

**S4 Table. Predictive factors for mortality among patients with AD without ACLF**

|  | 30 days | | | | 90 days | | | |
| --- | --- | --- | --- | --- | --- | --- | --- | --- |
|  | Univariate analysis | | Multivariate analysis | | Univariate analysis | | Multivariate analysis | |
|  | p value | OR (95%CI) | p value | OR (95%CI) | p value | OR (95%CI) | p value | OR (95%CI) |
| Age | 0.030 | 1.02 (1.00-1.04) |  |  | 0.064 | 1.02 (1.00-1.04) |  |  |
| Male sex | 0.066 | 1.13 (0.66-1.94) |  |  | 0.774 | 1.08 (0.65-1.80) |  |  |
| Presence of DM | 0.062 | 0.50 (0.24-1.04) |  |  | 0.201 | 0.66 (0.34-1.25) |  |  |
| Bacterial infection | 0.271 | 1.34 (0.80-2.67) |  |  | 0.049 | 1.65 (1.00-2.73) | 0.154 | 1.47 (0.87-2.50) |
| Laboratory information | | | | | | | | |
| Na | 0.982 | 1.00 (0.96-1.04) |  |  | 0.099 | 0.97 (0.93-1.01) |  |  |
| Hemoglobin | 0.436 | 1.02 (0.97-1.08) |  |  | 0.393 | 1.03 (0.97-1.09) |  |  |
| WBC | 0.284 | 1.00 (1.00-1.00) |  |  | 0.225 | 1.00 (1.00-1.00) |  |  |
| PMN% | 0.158 | 1.02 (0.99-1.04) |  |  | 0.052 | 1.02 (1.00-1.04) |  |  |
| Platelet | 0.194 | 1.00 (1.00-1.00) |  |  | 0.382 | 1.00 (1.00-1.00) |  |  |
| INR | 0.395 | 1.34 (0.68-2.62) |  |  | 0.179 | 1.56 (0.82-2.99) |  |  |
| Creatinine | 0.270 | 1.51 (0.73-3.15) |  |  | 0.116 | 1.75 (0.87-3.50) |  |  |
| HCO3 | 0.132 | 1.04 (0.99-1.09) |  |  | 0.279 | 1.02 (0.98-1.07) |  |  |
| TB | 0.599 | 0.99 (0.94-1.04) |  |  | 0.780 | 0.99 (0.95-1.04) |  |  |
| AST | 0.405 | 1.00 (1.00-1.00) |  |  | 0.609 | 1.00 (1.00-1.00) |  |  |
| ALT | 0.289 | 1.00 (1.00-1.00) |  |  | 0.301 | 1.00 (1.00-1.00) |  |  |
| ALP | 0.130 | 1.00 (1.00-1.01) |  |  | 0.074 | 1.00 (1.00-1.01) |  |  |
| Albumin | 0.373 | 0.90 (0.70-1.14) |  |  | 0.107 | 0.72 (0.48-1.07) |  |  |
| Lactate | 0.984 | 1.00 (0.89-1.12) |  |  | 0.234 | 0.93 (0.83-1.05) |  |  |
| Renal dysfunction | 0.424 | 1.26 (0.72-2.21) |  |  | 0.138 | 1.50 (0.88-2.56) |  |  |
| Prognostic scores | | | | | | | | |
| CLIF-AD score | 0.194 | 1.02 (0.99-1.04) |  |  | 0.024 | 1.03 (1.00-1.06) | 0.112 | 1.02 (1.00-1.05) |
| CLIF-OF score | 0.240 | 1.14 (0.92-1.42) |  |  | 0.252 | 1.13 (0.92-1.39) |  |  |
| CTP score | 0.102 | 1.10 (0.98-1.23) |  |  | 0.024 | 1.14 (1.02-1.27) | 0.055 | 1.12 (1.00-1.26) |
| MELD score | 0.551 | 1.01 (0.97-1.06) |  |  | 0.076 | 1.04 (1.00-1.08) |  |  |
| MELD-Na score | 0.872 | 1.00 (0.97-1.04) |  |  | 0.053 | 1.03 (1.00-1.06) |  |  |
